# The meaning and validation of social support networks for close family of persons with advanced cancer

**DOI:** 10.1186/1472-6955-11-17

**Published:** 2012-09-17

**Authors:** Catarina Sjolander, Gerd Ahlstrom

**Affiliations:** 1 The School of Health Sciences, Jönköping University, Box 1026, SE–551 11, Jönköping, Sweden; 2 The Ryhov County Hospital, SE–551 85, Jönköping, Sweden; 3 The Swedish Institute for Health Sciences, Department of Health Sciences, Lund University, Box 187, SE–221 00, Lund, Sweden

**Keywords:** Family members, Cancer, Social support, Social network, Confirmation, Latent content analysis, Walker and Avant concept analysis

## Abstract

**Background:**

To strengthen the mental well-being of close family of persons newly diagnosed as having cancer, it is necessary to acquire a greater understanding of their experiences of social support networks, so as to better assess what resources are available to them from such networks and what professional measures are required. The main aim of the present study was to explore the meaning of these networks for close family of adult persons in the early stage of treatment for advanced lung or gastrointestinal cancer. An additional aim was to validate the study’s empirical findings by means of the Finfgeld-Connett conceptual model for social support. The intention was to investigate whether these findings were in accordance with previous research in nursing.

**Methods:**

Seventeen family members with a relative who 8–14 weeks earlier had been diagnosed as having lung or gastrointestinal cancer were interviewed. The data were subjected to qualitative latent content analysis and validated by means of identifying antecedents and critical attributes.

**Results:**

The meaning or main attribute of the social support network was expressed by the theme *Confirmation through togetherness*, based on six subthemes covering emotional and, to a lesser extent, instrumental support. Confirmation through togetherness derived principally from information, understanding, encouragement, involvement and spiritual community. Three subthemes were identified as the antecedents to social support: Need of support, Desire for a deeper relationship with relatives, Network to turn to. Social support involves reciprocal exchange of verbal and non-verbal information provided mainly by lay persons.

**Conclusions:**

The study provides knowledge of the antecedents and attributes of social support networks, particularly from the perspective of close family of adult persons with advanced lung or gastrointestinal cancer. There is a need for measurement instruments that could encourage nurses and other health-care professionals to focus on family members’ personal networks as a way to strengthen their mental health. There is also a need for further clarification of the meaning of social support versus caring during the whole illness trajectory of cancer from the family members’ perspective.

## Background

Cancer care has to a greater extent come to be carried out at home. The need for such care is often sporadic at the outset, but tends to become more intense as the illness evolves. Taking on the new role and responsibility as caregiver, the family member must deal at the same time with the sudden onset of cancer and its potentially life-threatening nature [1-3]. In the early stage of cancer, the emotional reaction to a loved-one’s illness can be of a chaotic nature. Research has shown that there can be even greater risk of psychological distress for the family than for the patient [4], and the families of cancer patients can exhibit symptoms such as sleeplessness and depression [5,6]. Furthermore, family members may have to fulfill major life demands besides caring, for example holding down a job and childcare [7]. Those family members who do not have access to personal support or a social network are at high risk for depression [8,9].

Most research on the families of persons with cancer has focused on breast or prostate cancer, rather than cancers with a worse prognosis [10,11]. The scant literature on the families of lung or gastrointestinal cancer patients deals with the role of the caregiver and the risk (and predictors) of burden and depression. The results show a particularly high risk of emotional burden and psychological distress [12-14].

Social support is increasingly identified as helping people adjust to a stressful life. However, despite the large amount of empirical research on such support conducted in health care in the last 20 years, the findings remain inconsistent. This inconsistency can be attributed to the use of different measures and different operational definitions of the term social support from one study to the next [15,16]. Finfgeld-Connett conducted a study [17] to clarify the concept of social support by using findings from three linguistic concept analyses and 44 qualitative studies. The concept analysis revealed that a common way for nurses to view social support is as emotional and instrumental support [17]. Emotional support consists of comforting behaviours, which are intended to alleviate uncertainty, anxiety, hopelessness and depression. Instrumental support consists of providing tangible goods and services such as transportation and assistance with household tasks [17]. Another way of defining social support, commonly used outside of the caring context, is to make a distinction between structural and functional support [18]. Structural support implies a network of interpersonal relationships, involving relatives, friends and co-workers, through which the person is attached to his or her community. Functional support is usually described in terms of the provision of information, tangible support and emotional support [16-19]. Besides inconsistencies in descriptions of social support, the similarities between caring and social support can also be confusing in the literature [20]. Caring and social support have comparable attributes and both concepts are characterised as dynamic interpersonal processes directed towards improved mental well-being. In contrast, physical well-being is commonly only an outcome of caring when social support is connected mainly with the nonprofessional area [17,20].

A support network can be a resource for family members in a time of crisis [21-24]. There is a need for further research regarding the presence of social support networks for the families of persons in the early stage of treatment for advanced lung or gastrointestinal cancer. Such research will contribute to a better understanding of the family members’ need for support in a drastically changing life situation. There is at the same time a need to investigate what the term social support is taken to imply. Despite ubiquitous use of the term by both lay people and professionals, there is still a lack of clarity about its meaning, and social support is easily confused with, for instance, caring [17]. Against this background, the main aim of the present study was to explore the meaning of social support networks for close family of adult persons in the early stage of treatment for advanced lung or gastrointestinal cancer. An additional aim was to validate the study’s empirical findings by means of the Finfgeld-Connett conceptual model for social support. The intention was to investigate whether these findings were in accordance with previous research in nursing. The term “family” is taken to include more than just biological relatives or people related by marriage, referring instead to people identified by the patients as playing a key role in their lives [25].

## Methods

### Design

The present study is part of a larger prospective, longitudinal project initiated in order to acquire enough knowledge to construct and test an evidence-based intervention programme for easing family members’ situation and thus indirectly helping the patients. The study design is inductive-deductive, whereby latent content analysis of qualitative interviews has been combined with validation of the findings by means of a theoretical work based on metasynthesis [17]. Informed consent was obtained from all participants prior to the study. The family members were given written information covering the purpose of the study, the voluntary nature of participation and their freedom to withdraw whenever they wished. Confidentiality was assured, which means that findings cannot be linked to the individual. Ethical approval was granted by the Regional Ethical Review Board of the University of Linköping, Sweden.

### Selection of the participants and the study group

Family members included in this study had a relative who 8–14 weeks earlier had been diagnosed as having cancer in the lung or gastrointestinal area. The inclusion criteria were the following. Approximately half of the participants should be close family of persons with lung cancer, the other half close family of persons with gastrointestinal cancer; they had to be 18 years of age or older; and they had to be able to speak Swedish. The purpose of the first of these criteria was to obtain variations in data.

In- or out-patients who had been diagnosed as having lung or gastrointestinal cancer and who fulfilled the inclusion criteria were identified by nurses and physicians at one medical and two surgical clinics of two hospitals in the south of Sweden. These patients were given written and oral information about the study by the nurses and physicians and were asked whether they were willing to give the name of a family member who might be interested in participating in the study. Those who said yes were given a letter to hand over to the chosen family member, containing information about the purpose and design of the study and asking about participation. Family members who gave their written consent to participate were contacted by phone by the first author to arrange a time and place for the interview. Included in this study were the first 17 consecutive family members who agreed to participate. Background characteristics of the study group and patients are shown in Table 1.

**Table 1:** Background characteristics of the family members in the study group and the patients

	**Family members**	**Patients**
	**All (n = 17)**	**All (n = 17)**
	**n (%)**	**n (%)**
***Family members’ characteristics***		
**Age**		
Mean years (range)	56 (31–77)	
Up to 65 years	9 (53)	
From 66 years	8 (47)	
**Gender**		
Female	13 (76)		
Male	4 (24)		
**Own children**		
Small children or teenagers	6 (65)		
Adult children or grandchildren	11 (35)		
**Living situation**		
Sharing household with the person with cancer	10 (59)		
Separate household	7 (41)		
**Relationship to the person with cancer**		
Partner (five wives, three husbands)	8 (47)		
Cohabitant	2 (12)		
Grown child (four daughters, one son)	5 (29)		
Other relative (one ex-partner, one uncle)	2 (12)		
**Education**		
Upper secondary and above	10 (59)		
Less than upper secondary	7 (41)		
**Work status**			
Currently working	11 (65)		
Retired	4 (23)		
Student	1 (6)		
On sick leave	1 (6)		
***Patients’ clinical characteristics***			
**Age**		
Mean years (range)		65 (35–88)
Up to 65 years		12 (71)
From 66 years		5 (29)
**Gender**		
Female		7 (41)
Male		10 (59)
**Type of cancer**		
Lung		10 (59)
Pancreas		5 (29)
Stomach		2 (12)
**Cancer treatment**		
On-going chemotherapy		17 (100)
Radiation treatment		1 (6)
Surgical therapy #		2 (12)

# One had undergone surgical therapy and one was in preparation for it.

### Data collection

One week before the interviews were conducted a mailed questionnaire, the first of five data collections over a one-year period, was sent to each participant. It concerned the estimation of the number of hours spent on informal caregiving, the type of support given and the caregiver’s health and health-related quality of life. The background data in Table 1 are from this data collection which was completed within one week before or after the interviews. The other data have been published [26] or will be reported in further papers.

The interviews were about two areas, social support networks and management strategies; the latter area is covered in a published paper [27]. Three main open-ended questions regarding the family members’ views on social support networks in relation to their cancer experience were asked: (a) What does the term “support” mean to you? (b) Who are the persons who make up your support network? (c) What personal support have you received? Follow-up questions were asked to clarify and enrich the information given, for example Can you tell me more about the support you received? Can you tell me more about the people in your social support network? The duration of the interview was 60–90 minutes, the average being 75 minutes. The interviews were tape-recorded and transcribed verbatim. Seventeen interviews were included in the study then data saturation had been reached. This is based on that no new information emerged from the last three interviews. Nine of the seventeen interviews were carried out at the hospitals, six in the family members’ homes and two at their place of work.

### Data analysis

The background data are presented through descriptive statistics in Table 1. The interviews were subjected to qualitative latent content analysis. Both qualitative and quantitative content analysis imply the systematic reduction and transformation of a message into data (often text) such as can be communicated to other persons. Content analysis has come into wide use in studies within health care in recent decades and comprises a family of analytical approaches. Qualitative content analysis is divided in the literature into latent and manifest content analysis. Latent content analysis implies encoding and interpretation of the underlying meaning of the text, manifest content analysis implies encoding to descriptions without the intention of interpretation of underlying meaning [28-31]. Latent content analysis is applied in the present study. It constitutes an inductive approach making it possible to “listen to the words” of the text and acquire a better understanding of the participants’ perspective [28,29]. The analytical procedure used is shown in Table 2, and this procedure has previously been described by Graneheim & Lundman [29]. 

**Table 2:** Exemplification of the analytical procedure from the meaning units to the subthemes

**Meaning unit**	**Condensed meaning unit**	**Code**	**Subtheme**
“If there’s a problem during the day when my wife’s bad, I can just ring our daughter and she’ll get off work and come at once, which means I’ve got good support at home. We talk often, and she can sometimes give me good advice about what to do.” (Interviewee 2)	If there is a problem he rings their daughter, who can get off work and come at once. This is good support at home. They talk and she gives him good advice.	The daughter is on hand to give her father support and advice.	Understanding and support from relatives
“I’ve got my parents nearby, thank goodness. When my husband’s in hospital they come and help me every evening so that I can go and see him. Without my parents, I don’t know what would’ve happened about all the practical things at home. They help me put the children to bed in the evening so that I can go to the hospital. I could do with that help all the time.” (Interviewee 10)	Has her parents nearby. When her husband is in hospital they come and help her put the children to bed every evening so that she can go and see him. She doesn’t know what she would have done without them. They help with the practical things at home.	Appreciates her parents’ help with home and children.	Understanding and support from relatives
“As soon as I feel distressed my sister and other people close to me come over at once. It’s pretty odd, really. The people I feel I get most support from are the ones who’ve been through crises of their own and have had a bit of a hard time themselves. My sister’s husband’s ten years older and he’s got skin cancer, so in a way I support her too.” (Interviewee 7)	As soon as she feels distressed her sister and other people close to her come over. Finds it odd that the people she feels give her most support are those who have experienced crises of their own and are themselves in a difficult situation.	Wonders about the fact that she gets most support from those who have experienced crises of their own.	Greatest understanding from those who have experienced crises of their own
“I’m alone here. He’s got a son. He’s too immature to handle a loss so he avoids coming here. It makes me very sad, the way he mostly rings and makes some excuse. They like each other, it’s not that. Dad and son like each other but the way I see it he’s too immature to bear the sorrow.” (Interviewee 3)	Alone in the situation. Husband’s son is too immature to bear the sorrow. This makes her very sad, because father and son like each other very much. The son often rings with some excuse.	Observes with sorrow how the son can’t cope with meeting his father.	Desire for a deeper relationship with relatives
“Our employer’s just fantastic. He always stops to chat with whoever he meets. Twice he’s taken the time to visit us at home. It gives you such a sense of security.” (Interviewee 7)	Employer just fantastic. He always stops and talks. Has twice taken the time to visit. This gives such a sense of security.	Admires and appreciates her employer who has visited her at home.	Involvement of fellow-workers and employer

Initially the audio-taped interviews were listened to and read through several times to obtain a sense of the whole content. Thereafter, each interview was divided into meaning units, i.e. sentences or paragraphs that related to the same central meaning [29]. These units were then condensed, which is to say they were shortened but retained their core content. The condensed meaning units were then abstracted and labeled with codes. From these codes, which were constantly compared and contrasted with reference to the meaning units and the interview text, subthemes emerged after a thorough analysis (Table 2). The subthemes were then related to each other and again scrutinised to verify their relevance. Finally, through a careful process of comparison between codes and subthemes (including reference to the meaning units), there emerged the theme *Confirmation through togetherness*, which was related to all the subthemes and expressed the main thread or main latent content of the text.

#### The validation procedure

The validation procedure was performed in two steps. Firstly, the trustworthiness of the analysis was guaranteed by means of an independent analysis by the second author (GA). Having read all the interviews to obtain a sense of the whole, this author examined the meaning units, condensed meaning units, codes and subthemes in order to detect any bias in the primary analysis made by the first author (CS). For the sake of trustworthiness, comparisons were made between the analyses performed by CS and GA, and any discrepancies were resolved through discussion. These comparisons focused on similarities and differences of content at every stage of the analysis.

Secondly, when the inductive analysis was completed, the theme and subthemes were compared with the Finfgeld-Connett conceptual model [17] to highlight the similarities and differences of content. The Finfgeld-Connett metasynthesis was based on studies published from 1987 to 2003 [17]. The analysis involved use of a matrix organised in accordance with that of Walker and Avant [32,33], i.e. identifying antecedents (preceding occurrence, cause or event), critical attributes of the study in focus and consequences/outcomes. This matrix was also applied to the findings of the present study for the sake of comparison. The comparison started with several thorough readings of what is set forth in the Finfgeld-Connett model [17] concerning the meaning of the different concepts of social support, with application to our findings [33]. The meaning units of the different subthemes were scrutinised in order to classify the subthemes not only in terms of antecedents and attributes, but also in terms of emotional support and instrumental support. A great effort was then made to judge similarities and differences in the findings of the present study as compared with Finfgeld-Connett’s metasynthesis [17].

## Results

**Figure 1:**
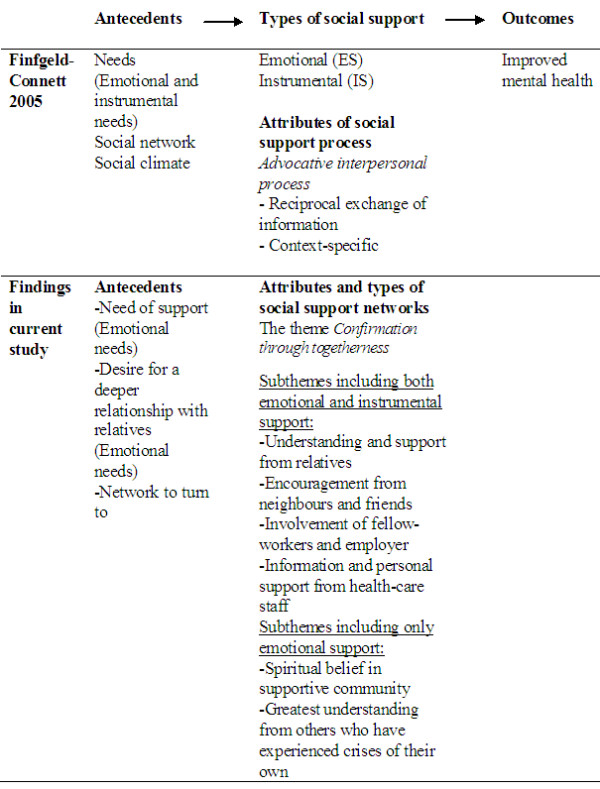
Comparison between the meaning of support for family members in this study and the findings of the metasynthesis set forth by Finfgeld-Connett (2005).

The participants’ descriptions resulted in one theme *Confirmation through togetherness*, and nine subthemes: (1) Need of support, (2) Network to turn to, (3) Understanding and support from relatives, (4) Encouragement from neighbours and friends, (5) Greatest understanding from those who have experienced crises of their own, (6) Involvement of fellow-workers and employer, (7) Spiritual belief in supportive community, (8) Information and personal support from health-care staff, and (9) Desire for a deeper relationship with relatives (see Table 3). The most frequent subtheme was Understanding and support from relatives. In the following text the theme and subthemes are described and sorted in accordance with the Finfgeld-Connett model (Figure 1). Three of the subthemes were classified as antecedents of social support, the other six as attributes of it; and this support is divided into two types, namely Emotional support (ES) and Instrumental support (IS). Each subtheme is illustrated through two quotations to clarify the meaning of social network.

**Table 3:** Frequencies of meaning units per subtheme generating the theme Confirmation through togetherness

**Subthemes**	**Meaning units**
	**Number (%)**
Understanding and support from relatives	86 (32)
Encouragement from neighbours and friends	49 (18 )
Need of support	30 (11)
Network to turn to	29 (11)
Desire for a deeper relationship with relatives	23 (8)
Information and personal support from health-care staff	18 (7)
Involvement of fellow-workers and employer	17 (6)
Spiritual beliefs in supportive community	11 (4)
Greatest understanding from those who have experienced crises of their own	9 (3)

### Antecedents of Social Support

#### Need of support

The “Need of support” was apparent when the family members spoke about their experiences of overwhelming despair when faced with someone in their family being diagnosed as having advanced cancer. They expressed a great deal of psychological distress in the form of worry and sadness regarding the uncertain prognosis and the adverse effects of aggressive treatments such as chemotherapy. All were aware of the threat of death implied by the advanced cancer diagnosis.

"*I try to keep in good spirits and not think about my partner’s illness and what the future’s going to be like. Deep down, it upsets me terribly that my partner’s not in good health. (Interviewee 3)*"

"*I was supposed to be seeing a psychologist but there were complications and it didn’t happen. I certainly could have done with seeing someone I could talk to more often. I had a very short chat with someone at work, a woman who put me in touch with the psychologist I was going to be talking to. But, as I say, it hasn’t happened yet. (Interviewee 12)*"

#### Desire for a deeper relationship with relatives

Family members did not always find social support to be adequate. There had in many cases been more frequent, closer contact before the cancer, and they wanted this contact restored. Some participants felt constantly rebuffed when they sought to maintain the relationship as it was previously. It was difficult to cope with relatives’ inability to deal with the situation and keep the relationship unchanged. Family members felt that their relatives did not always assign high priority to meeting them. The excuse offered was lack of time, and relatives gave the impression of having a lot to do managing their own lives. There was the feeling that relatives could not face encountering the person with cancer and the family member’s sorrow at that. Some relatives were not capable of providing the support the family members needed and wanted because they had problems of their own. Nevertheless, family members did perceive concern on the part of their relatives, even though the latter could not always put this concern into words.

"*There’s no help in the sense that there’s a let-up for me. You talk to your relatives but it doesn’t go beyond that. Some of them think it’s a trying situation. (Interviewee 2)*"

"*Our daughter-in-law’s nice, but she’s not very considerate. But one Saturday I was talking to her on the phone and then on the Monday I found a bunch of flowers hanging on the door-handle... My daughter-in-law does care, but she’s not as open as I am. But of course they’re in the midst of their own lives and they’ve got a child. (Interviewee 7)*"

#### Network to turn to

Family members spoke of having an existing network, often established long before the diagnosis. None spoke of any attempt to build a new networks during the last few months. They felt that they already had a personal network to turn to, sometimes more than one. The network was of particular value to them now that they were under such great emotional pressure. They derived a great sense of shared reality from being able to turn to these people whenever they wanted to, without having to make arrangements in advance. Sometimes it was a relief just to be with someone and to give expression to the sorrow they felt. In certain cases there was a deeper contact, one of particular importance. Having especially close friends to turn to meant a great deal, and gave an increased sense of not being alone with one’s feelings.

"*One day of the week, we either talk over the phone or go somewhere and talk, to pass the evening. But they always ring. One of them rang yesterday to see how things were. We didn’t just talk about the illness, we talked about other things too. We go there when we feel like it. It’s people we’ve known for years. (Interviewee 13)*"

"*We’ve got ever such a lot of friends and acquaintances, 30 or 40. There are six couples we see most. They’re the ones I turn to; the ones me and my husband are really close to. (Interviewee 14)*"

#### Comparison with antecedents in the Finfgeld-Connett metasynthesis

The two subthemes Need of support and Desire for a deeper relationship with relatives were linked closely to the antecedent Needs in the model presented by Finfgeld-Connett [17] as depicted in Figure 1. However, these two subthemes in the present study included only emotional needs, whilst the model also identifies instrumental needs such as tangible goods, transportation services and financial support. The metasynthesis [17] revealed that social support requires that the person himself/herself shall perceive a need for support and accept this need, which is closely related to the person’s perception of his/her ability to handle the current situation. In addition, the potential providers of support must recognise the need and be willing and able to offer the support [17]. The findings of the present study established that the family members were aware of their need of support but felt that not all of their relatives were ready to provide such support. However, the third antecedent in the model, “Social climate,” [17] was only implicitly embedded in the subtheme Network to turn to. “Social climate” may be most helpful if the persons involved have a shared context where one or more persons can anticipate, interpret and respond to a person’s need [17].

### Attributes of social support

#### Confirmation through togetherness

The inductive content analysis resulted in the emergence of one theme from the six subthemes described below. Confirmation through togetherness is a question of being confirmed as a person in this new situation of having a close relative with cancer — confirmed through a feeling of togetherness. The contact that family members had with their social networks made them feel that they were active members of a community. They derived strength from the feeling of belonging, and from the sympathy and feeling of fellowship they encountered. This support and solicitude gave them a sense of security. Their social networks offered warmth and involvement, without their needing to ask for it. The sense of belonging gave them confirmation as persons, and confirmed the meaningfulness of their role as the person close to a person diagnosed as having cancer. This made it easier for them to manage this new life they were living.

#### Understanding and support from relatives

Family members felt strongly supported by others in their immediate and more distant family. These relatives provided practical assistance with things like child-minding, housekeeping and gardening, and it was a great relief not to have to worry about these things. Participants felt it was essential to have their closest relatives near at hand. They needed people to talk to, people who would listen. There was a sense of security in being understood, supported and helped without always having to ask. Their family was there for them. Being able to talk to family and openly show feelings was liberating and brought emotional relief. There was a feeling of togetherness when sorrows were shared and family members felt that they could be themselves. Often it was enough just to phone someone in the family and get things off your chest; this could mean just as much as meeting face-to-face. Being able to phone your child, brother or sister, for instance, made it so much easier to manage. It was seen as a good thing to have someone to share experiences with, someone who listened and was clearly involved.

"*My brother’s more down-to-earth and practical. When I’m upset about something and ring him, he can always calm me down. But my sister cries with me instead — which is good when all I want to do is cry, which sometimes happens. Sometimes I need calming down, and then I ring him. So they’re obviously a big help, both of them in their different ways. (Interviewee 4)*"

"*I’ve got three girls, and it’s the eldest I’m most in touch with. My grandchildren phone me. I know they think of us and feel involved. It means everything; I don’t know whether I could’ve coped otherwise. We may see things differently, but I need to talk and that’s what I’m able to do. There’s always someone to listen and wonder how things are. It means an enormous amount. (Interviewee 9)*"

#### Encouragement from neighbours and friends

In their difficult situation, family members found that regularly chatting to neighbours was important to them. An encouraging word from a neighbour had a calming effect, and knowing that they could count on their neighbours gave them a sense of reassurance and lightened the distress. That neighbours wanted very much to be easy to reach when the family member needed them, and the concern they expressed about the family member’s well-being gave participants a sense of being embraced by a community of friends. Family members felt that their neighbours and friends truly desired to provide all the help and support they could. They often got in touch spontaneously and wanted to show their involvement when, for instance, the sick person was undergoing arduous treatment. The neighbours’ and friends’ emotional and practical support enriched the family member’s daily life and made it easier to endure the trials of having cancer in the family.

"*Neighbours help when it comes to taking the dog out, getting in firewood, getting the fire started and so on. They come round spontaneously and ask if there’s anything they can do to help. When he was so weak, because he was going for treatment, they offered to help right away. (Interviewee 6)*"

"*We’ve got a lot of neighbours out here in the country, and they take a great interest in my welfare. It’s mostly whole families we’ve spent time with since the children were grown-up. They’re still our friends. There are some you never lose touch with and that you feel you’ve known all your life. They’re so encouraging and always looking on the bright side. (Interviewee 8)*"

#### Involvement of fellow-workers and employer

Family members felt they had support at their workplace in that their fellow-workers and employer showed concern and involvement. Chatting to, or simply being with, fellow-workers who were aware of the situation gave family members the chance to forget their troubles for a while. Fellow-workers showed understanding and consideration. They were aware that family members could not always work as efficiently as before and stood in for them when they needed to go to the hospital with the sick relative. In some cases, the employer adjusted the family member’s work schedule in periods of crisis. It was an advantage for family members that the people at work knew the state of affairs and knew that the quality of the family member’s work might vary from day to day, depending on how they felt.

"*They understood completely; they knew why I wasn’t always on form. I could have down-periods and didn’t have any energy. They knew about that sort of thing; they knew just what I was going through. So I’m glad I was at that place. (Interviewee 10)*"

"*The man I work with is wonderful. We talk. We have great fun at work. It feels like he’s a really close friend. I like talking a lot, and when he’s not there it feels empty and lonely, and then I brood a lot. Sometimes I cry, but he accepts that and comes and gives me a hug. We can talk about it too. (Interviewee 11)*"

#### Spiritual belief in supportive community

Family members found it supportive to go to a Christian church and take part in church activities. Going to church and being with their friends from church gave them another opportunity to talk about their situation, to be met with understanding and receive emotional support. Regularly meeting people at church gave them a sense of spiritual belief and community. The experience of sharing this belief with their social network from church, the experience of being together in this spiritual community, gave them relief and consolation. The presence of friends, the minister, the religious community and the existential conversation meant that the heavy yoke was not borne alone. The grief over the impending loss of the loved-one became more bearable through being a member of this community and through the perception of being in communication with God. Faith in the resurrection meant that the family member and the person with cancer would be reunited one day in heaven. This helped family members to endure such things as the change in appearance of their loved-one as a result of hair loss from radiation therapy. Encountering other people through the church and prayer strengthened family members’ faith.

"*We go to the church, and we’ve got a broad network there. We get a lot of support, both my husband and I. (Interviewee 1)*"

"*They’re there, of course, as friends giving you support. I mean it’s part of our faith. God has given me the chance to talk about things. I can talk to Him in prayer.. My faith and all the contact with others, that’s where I’ve found relief, that’s what’s meant I don’t have to bear the heavy yoke all on my own. (Interviewee 17)*"

#### Greatest understanding from those who have experienced crises of their own

Family members experienced the greatest sense of community in relation to people in their social network who themselves had experienced having a person with cancer in the family. The support received from these people was invaluable in that they knew what it meant to be in such a position and were therefore able to understand completely. It was comforting to be able to share sorrow with a person who had been through a similar experience. It was not always necessary to talk; it could be enough simply to know that the other person understood. The understanding offered by people who had experienced similar crises gave family members the strength to cope, and it was easier to express feelings in the presence of such people.

"*She knows how things are. I can call her up, go to her and cry if I want to; she’s there for me. She knows how it is with Dad and everything. She herself had a mother who was seriously ill. She knows what it’s all about. I don’t need to say anything; she doesn’t need to say anything, but we know. We can ventilate the situation and then talk about something else. It’s good to know that you can go to someone on the outside. (Interviewee 15)*"

"*He’s a great support. Four years ago his dad died of cancer, so he knows what it feels like. He’s ready to help and he comes along and backs me up when I take Mum to the hospital. It’s mostly a question of being able to talk. His support helps lower the pressure, helps me get my feelings out into the open. (Interviewee 16)*"

#### Information and personal support from health-care staff

Family members felt a sense of participation when health-care staff spoke to them directly. They appreciated being given information about the diagnosis, prognosis and treatment. It was also important to know where to turn for information. Family members received a file from the nurses with information about the community home-help service. They derived a sense of security from knowing that they could get answers to their questions from both the doctor and other health-care staff. It was felt to be important that staff should provide information in a manner that showed they cared and that information and support should be addressed directly to family members.

"*I’m in touch with a psychologist I can talk to — it’s been a great help. Being able to talk to somebody about these things makes it easier and it’s a great relief. (Interviewee 5)*"

"*The staff are fantastic. They talk to me a lot, both the doctor and all the others. They say I’ve got to think of myself and then I’ll be able to cope. We talk, I suppose that’s all you can do; I don’t expect more. (Interviewee 9)*"

#### Comparison with attributes of social support in the Finfgeld-Connett metasynthesis

The designation of the theme contains two keywords. Confirmation is usually expressed in the literature as affirmation or validation, whilst togetherness is not identified in the metasynthesis at all [17]. Three of the keywords of the subthemes (information, encouragement and spiritual beliefs) are to be found in the metasynthesis as attributes of social support [17]. The metasynthesis shows that social support involves the reciprocal exchange of verbal and/or nonverbal (flowers, cards, eye movements, facial expressions) information that is characterised by advocacy. Information consists of facts, advice, words of reassurance, positive affirmation and empathy. Encouragement is a common advocative strategy employed in an unconditionally positive atmosphere. Spiritual belief systems are sometimes considered to be part of social support networks but authors provide little explanation regarding this phenomenon [17]. However, there were two keywords of the subthemes that were not to be found in the metasynthesis, namely understanding and involvement.

#### Outcomes in improved mental health in the Finfgeld-Connett metasynthesis

According to the metasynthesis [17] the outcome of social support in the broad sense of improved mental health is in large part a question of an enhanced sense of personal competence, experiences of empowerment and an enhanced sense of reassurance. Involved here are feelings of well-being and diminished distress.

## Discussion

The findings of this study have identified the significance of the social support network for family members in the early period after a close relative has been diagnosed as having lung or gastrointestinal cancer. Our findings verify the definition of social support as a reciprocal exchange of verbal and/or nonverbal information [17]. The main finding in the inductive analysis was expressed through the theme Confirmation through togetherness, which entails being seen and confirmed as a person in one’s new situation of having a loved person with life-threatening cancer in the family, and being respected for one’s experience and knowledge of the sick person’s life situation. The participants indicated that strength was derived from togetherness, from a sense of being an active member of a community and encountering sympathy. The relationship as member of a community is interpreted as more equal than where there is an advocative interpersonal process [17]. The predominant providers of support in the case of the present study were mainly lay persons. Health-care professionals are considered as support when lay persons cannot provide the support that is required [17], and in this case the advocative interpersonal process may be common.

Validation of our findings through the previous metasynthesis revealed that half of the meanings of social support networks are already well-known from previous qualitative research [17] (i.e. confirmation, information, encouragement and spiritual beliefs) whilst togetherness, understanding and involvement were not described. One explanation of the discrepancy between these results is that most of the studies in cancer care are based on descriptions of need of social support or interventions from the perspective of professionals [17,34]). However, the present study verifies that social support is provided mainly by non-professionals, and this may be one reason for the additional meanings found in the study. Another possible explanation is that social support is context-specific and previous research has mainly focused on patients’ need of social support as part of caregiving [17] whilst the present study focuses on the social support family members receive mainly from lay persons.

The comparison of the findings of our study with the metasynthesis study by Finfgeld-Cornett is a response to existing inconsistent findings in the literature about social support which limit its usefulness in nursing [17]. In nursing science there is also need for more research that can inform practice, and when the issue is complex it is necessary to carry out a chain of studies with different designs before recommendations for practice can be made [17]. During the last five years there have been an increased number of interventions for informal caregivers in cancer and palliative care. It is necessary to continue to focus on mechanisms of intervention, tightly focused aims and outcomes, robust designs and a plurality of models and target populations/settings [34]. Our findings show that networks of significant others (relatives, employer, fellow-workers, etc.) are experienced as meaningful by family members and give them confirmation in their distressing life situation. Characteristic of this situation is that the family member is helping the patient through informal caregiving whilst at the same time trying to prepare themselves for the person’s eventual death [35]. Confirmation lightened the distress and made it easier to deal with the situation [36-38]. Finfgeld-Connett [17] established that the need for social support has a psychosocial substratum, which was verified in the present study through the antecedents Need of support and Desire for a deeper relationship with relatives. Family members also spoke, though less often, of receiving help with practical matters, which spared them additional distress.

Several steps are needed in the chain of studies [39] about social support networks. One is to examine nursing intervention to bolster existing networks or to promote the development of new ones [17]. Another step is to develop instruments for use in the evaluation of non-professional social support based on Confirmation through togetherness and the main attributes. Developing measurement instruments [17] from this expanded conceptual model could encourage nurses and other health-care professional to focus on family members’ personal networks as a way to strengthen mental health. Nurses should reconsider social support as a part of nursing intervention and differentiate social support from concepts such as caring [17].

In the present study, family members appreciated support from neighbours; it gave emotional relief and strengthened their ties to these neighbours [40,41]. The findings also revealed that some family members desired a deeper relationship with relatives and wanted to talk to them more. Relatives could not always face their own and the family members’ sorrow. When the family members did not get the support they wanted from relatives, they felt alone and isolated [38]. This feeling of isolation was not experienced in relation to neighbours, fellow-workers, employers and health-care staff. Possibly family members had higher expectations of close relatives with regard to the providing of support.

In addition to the social support network, the person’s own activities to manage the distress are of importance for mental health [17]. A previous study within our larger research project revealed that being with other people is a way of distracting family members’ thoughts. They can think of something else for a while when in the company of friends and co-workers. They find temporary solace and escape from their worries [27]. However, it is important to distinguish between “*seeking* social support,” which is a theoretical construct of management or coping, and (simply) “social support,” which requires other persons’ willingness to participate in a mutual exchange with the person seeking the support. Management is based on the person’s own appraisal of the actual demands and effort to cope with the current problem [42]. Social support consists of the actions that others perform to assist a particular person [19,43-46]. The person’s capacity for coping with distress partly depends on the support he or she receives from the family and the social network [20,42,47].

Despite the fact that religious beliefs are more common in other countries than in Sweden [48], the family members in this study derived comfort from spiritual beliefs, through the sense of togetherness derived from sharing Christian belief with friends from church. A similar finding emerged from a study involving four interviews with 20 patients with inoperable lung cancer and their informal caregivers over a one-year period [49]. Many of the patients in their last year of life expressed spiritual needs involving seeking meaning and purpose in life. However, family members also had their own spiritual needs [49]. Incorporating spiritual well-being into health care is essential as existential diversity grows in globalised societies, which means that health-care staff need to be very aware of each patient and family member’s particular needs and must never view anything as just a matter of common sense [50]. When patients and family members were given the opportunity to discuss their spiritual needs with staff, they valued this greatly as it validated their concerns and made them feel cherished [49]. However, patients and family members were often reluctant to take the initiative in raising spiritual issues with “busy” staff. They did not see spiritual needs as directly relevant to the health-care professional’s role, for which reason they actively sought to disregard their spiritual distress [49]. The need for spiritual care was investigated in a study with 156 adult cancer patients and 68 family caregivers. The findings showed that some cancer patients and family caregivers are enthusiastic about receiving some form of spiritual care, whilst others do not want it [51]. However, religion and spirituality are two separate constructs, not interchangeable though sometimes overlapping. Religion, often centrally concerned with spirituality, is also a social phenomenon, characterised by social and cultural concerns and goals. Spirituality is a much broader construct than religion and the two constructs do not overlap for people who are spiritual but do not practise a religion, or indeed for people engaged in religious practices who are not spiritual [52,53]. Staff require knowledge of spiritual beliefs and spiritual caring, which also implies reflection on and awareness of their own beliefs [54]. Using a theoretical framework and guidelines can better prepare staff to incorporate spirituality into their practice [52,53].

Family members expressed a need for informational and personal support from health-care staff, which has also been found in previous research [55]. Family members feel confirmed as persons important for the patient if they are listened to and respected by staff [36,38]. An interesting finding from the present study is that participants found the most supportive persons to be other people with similar experiences. This is an aspect that could be integrated into interventions in health care to great advantage. Furthermore, some participants expressed a need for psychological support for close relatives who could not deal with someone in their family having advanced cancer. This indicates that staff need to apply a family system approach to assist the family [56]. This, in turn, underlines the importance of developing supportive interventions from a preventive perspective at an early stage of the illness trajectory. Research shows that the design of interventions directed towards family members should be based on the specific needs of these people. Support groups using the Internet as a forum to facilitate supportive communication are increasing in number [57,58]. Interactive web-based programs for cancer patients and their caregivers offer an opportunity to deliver tailored information in an efficient, accessible and cost-effective manner [59]. However, there is an urgent need to evaluate the implemented interventions using valid methods and study designs such as randomised controlled studies, as also to assess lay persons’ provision of social support from the family members’ perspective. At present there is only limited evidence of the effectiveness of support interventions [60].

### Methodological considerations

This study was limited to exploring the meaning of social networks for family members who are faced with having an adult relative with cancer in the early stage of treatment. Therefore the findings should only be transferred to family members in a similar context. Several limitations must be taken into account when interpreting the findings. One limitation with regard to the transferability of the findings is the small proportion of male participants (4 of 17 participants). The nurses who asked patients to participate were not predominantly male; the patients, however, were mainly male, and all except one chose a female family member to participate. In addition, it is possible that the family member chosen to participate was not the one most dissatisfied with their social support. Gratitude on the part of the family member regarding the possibility of the patients receiving curative treatment, as also deep respect for the health-care professionals’ commitment to helping the patient, may also have influenced the findings. Another limitation is that the data can be presumed to have low stability in that family members’ experiences probably change over time as the illness progresses. The family members in this study were not experiencing high caring demands, as the patients were in the early stage of the illness trajectory. A longer period of illness often means a greater amount of caregiving and as a consequence more isolation, both for the family member and for the patient [61]. Longitudinal research on a similar sample is required for the establishment of greater dependability.

Furthermore, the interview questions in our study were not designed to capture the features of antecedents and outcomes of social support, therefore — not unexpectedly — our findings were not strongly related to the features of the antecedent Social climate and the outcome Improved mental health. This must be kept in mind when comparing the results of the present study with the conceptual analysis of Finfgeld-Connett [17].

The strength of this study is the two steps of the validation procedure. The achievement of credibility in the inductive approach to qualitative content analysis implies careful consideration of issues arising at every stage of the analytical process [29]. In the present study the basic principles of latent content analysis were applied, which means that there was systematic coding into subthemes and then integration into a theme [29]. The credibility of the inductive analysis was strengthened by means of comparing the subthemes and the theme with concepts from 44 qualitative studies in the previous metasynthesis [29]. The findings added more attributes to the previous model, and a significant contribution is the insight that social support is provided mainly by non-professionals.

## Conclusions

Our study verifies that a social support network involves reciprocal exchange of verbal and non-verbal information. The providers were mainly lay persons and social support was abstracted into the theme Confirmation through togetherness. The family members felt themselves to be members of networks, indicating a more equal relationship than in a care relationship with health-care professionals. The networks contributed information, understanding, encouragement, involvement and spiritual belief systems. It is a question of one or more networks linking relatives, friends with and without similar experiences, neighbours, employer, fellow-workers, members of their spiritual community and health-care professionals. The findings of the study expand the previous conceptual model in nursing with regard to social support by presenting the family members’ perspective. Besides their mainly positive experiences of social support networks, the family members reported distress with regard to close relatives who shied away from the troublesome situation.

### Implications

Further research is needed to provide more clarification of the meaning of social support networks provided by lay persons and the meaning of caring by nurses because of new circumstances that occur during the illness trajectory of cancer. The findings underline the importance of, and need for, longitudinal research on the whole cancer trajectory, including repeated interviews, and quantitative surveys involving a larger population. In addition, the findings indicate the need to transfer the available knowledge in the form of a measuring instrument that is sensitive enough to evaluate nursing interventions for family members of adult persons with advanced lung or gastrointestinal cancer. Nurses and other health-care professionals need to encourage family members to use and enhance personal support networks, as they have a positive effect on coping and mental well-being.

## Competing interests

The authors declare that they have no competing interests.

## Authors’ contributions

CS and GA designed the study. CS conducted the interviews and the initial analysis of the interview transcripts. Each step of the analysis was then scrutinised and discussed by the authors, and both authors read and approved the final manuscript.

## Pre-publication history

The pre-publication history for this paper can be accessed here:

http://www.biomedcentral.com/1472-6955/11/17/prepub
